# Bacterial concrete: the future of self-healing and sustainable infrastructure

**DOI:** 10.1016/j.mex.2025.103569

**Published:** 2025-08-14

**Authors:** Anumol Sukumaran, V Johnpaul, N Balasundaram, S Senthil Kumar

**Affiliations:** a Department of Civil Engineering, Karpagam Academy of Higher Education, Coimbatore, Tamilnadu, India; b Department of Civil Engineering, Sreepathy Institute of Management and Technology, Palakkad, Kerala, India; c Department of Civil Engineering, KSR College of Engineering, Tiruchengode, Tamilnadu, India

**Keywords:** Bacterial concrete, Self-healing concrete, Durability enhancement, Bacillus subtilis, Sustainable construction

## Abstract

This study investigates the durability enhancement of bacterial concrete incorporating microbial strains (Bacillus Licheniformis, Bacillus Flexus, Pseudomonas stutzeri, Escherichia coli, and Bacillus subtilis) through microbial-induced calcium carbonate precipitation (MICP). Various durability tests, including water absorption, RCPT, sulphate resistance, hydrochloric acid strength loss, sorptivity, and energy-dispersive X-ray analysis (EDAX), were conducted to evaluate the effectiveness of bacterial concrete. Bacterial concrete significantly reduces water absorption and chloride ion penetration, with Bacillus subtilis (M16) and Bacillus Flexus (M7) demonstrating the highest impermeability. Sulphate resistance analysis confirmed reduced weight loss before and after healing, highlighting microbial self-healing capabilities. Hydrochloric acid strength loss and sorptivity tests further validated improved acid resistance and reduced capillary absorption. EDAX analysis confirmed the formation of calcium carbonate, contributing to matrix densification and enhanced durability. Overall, microbial concrete exhibited superior resistance to environmental degradation, with Bacillus subtilis, Bacillus Flexus, and Bacillus Licheniformis at higher concentrations (10^6^ cells/ml) providing the most significant improvements.

Bacterial concrete showed increased workability and notable compressive, flexural, and split tensile strengths with Bacillus subtilis and Bacillus licheniformis at 10⁶ cells/mL,

Bacterial concrete provide the best self-healing and strength recovery capability; SEM and XRD data revealed higher density and effective crack healing.

Bacterial concrete is a sustainable material since it provides long-term durability by means of inherent self-healing systems.

## Graphical abstract

**Figure:**
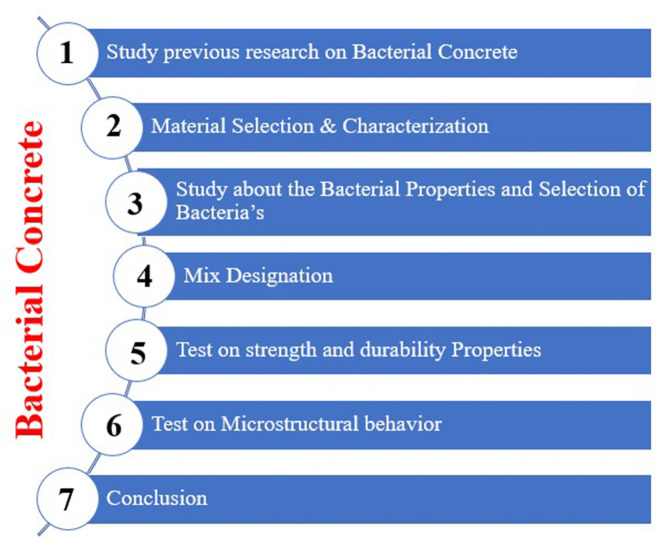

Specifications table

**Table:** 

**Subject area**	
**More specific subject area**	Civil Engineering
**Name of your method**	Bacterial Concrete with micro structural characterization
**Name and reference of original method**	If applicable, list the full bibliographic details of any key reference(s)that describe the original method you customized
**Resource availability**	• T. H. Nguyen, E. Ghorbel, H. Fares, and A. Cousture, "Bacterial self-healing of concrete and durability assessment," Cement and Concrete Composites, vol. 104, 2019, Art. no. 103340 [Online]. Available: https://doi.org/10.1016/J.CEMCONCOMP.2019.103340. • L. Chaurasia, V. Bisht, L. Singh, and S. Gupta, "A novel approach of biomineralization for improving micro and macro-properties of concrete," Construction and Building Materials, vol. 200, pp. 247–254, 2019 [Online]. Available: https://doi.org/10.1016/J.CONBUILDMAT.2018.11.031. • J. Akhtar, R. A. Khan, M. Akhtar, and J. K. Nejem, "Influence of natural zeolite and mineral additive on bacterial self-healing concrete: A review," Civil Engineering Journal, vol. 8, no. 5, pp. 815–830, 2022 [Online]. Available: https://doi.org/10.28991/cej-2022-08-05-015. • R. K. Bandlamudi, A. Kar, and J. R. Dutta, "A review of durability improvement in concrete due to bacterial inclusions," Frontiers in Built Environment, vol. 9, Art. no. 1095949, 2023 [Online]. Available: https://doi.org/10.3389/fbuil.2023.1095949. • F. Nosouhian, D. Mostofinejad, and H. Hasheminejad, "Concrete durability improvement in a sulphate environment using bacteria," Journal of Materials in Civil Engineering, vol. 28, no. 6, Art. no. 04015064, 2016 [Online]. Available: https://doi.org/10.1061/(ASCE)MT.1943-5533.0001337. • T. Priya, N. Ramesh, A. Agarwal, S. Bhusnur, and K. Chaudhary, "Strength and durability characteristics of concrete made by micronized biomass silica and bacteria-Bacillus sphaericus," Construction and Building Materials, vol. 198, pp. 570–577, 2019 [Online]. Available: https://doi.org/10.1016/J.CONBUILDMAT.2019.07.172. • M. Sarkar, D. Adak, A. Tamang, B. Chattopadhyay, and S. Mandal, "Genetically-enriched microbe-facilitated self-healing concrete – a sustainable material for a new generation of construction technology," RSC Advances, vol. 5, pp. 105363–105371, 2015 [Online]. Available: https://doi.org/10.1039/C5RA20858K. • V. K. R, R. Manju, and S. T., "Study on strength and durability properties of bacterial concrete," International Journal of Advanced Research in Science, Communication and Technology, vol. 2, no. 3, pp. 1–6, 2022 [Online]. Available: https://doi.org/10.48175/ijarsct-2227. • S. Luhar, I. Luhar, and F. Shaikh, "A review on the performance evaluation of autonomous self-healing bacterial concrete: Mechanisms, strength, durability, and microstructural properties," Journal of Composites Science, vol. 6, no. 1, Art. no. 23, 2022 [Online]. Available: https://doi.org/10.3390/jcs6010023. • A.Puram, "An experimental investigation on mechanical properties of bacterial concrete using Bacillus subtilis," International Journal for Research in Applied Science and Engineering Technology, vol. 12, no. 6, pp. 51–56, 2024 [Online]. Available: https://doi.org/10.22214/ijraset.2024.62338. • S. R. Vempada, M. S. Rao, S. Saduwale, P. Mahesh, T. V. Suneetha, and D. Nemova, "Pore structure characterization of bacterial concrete," MATEC Web of Conferences, vol. 392, Art. no. 01002, 2024 [Online]. Available: https://doi.org/10.1051/matecconf/202439201002. • Y. Dhandapani and M. Santhanam, "Investigation on the microstructure-related characteristics to elucidate performance of composite cement with limestone-calcined clay combination," Cement and Concrete Research, vol. 129, Art. no. 105959, 2020 [Online]. Available: https://doi.org/10.1016/j.cemconres.2019.105959.

## Background

Bacterial concrete, also known as self-healing concrete, is an innovative material that incorporates bacteria to enhance the durability and microstructural properties of concrete. This technology leverages the ability of certain bacteria to precipitate of CaCO_3_, which fills cracks and improves the structural integrity of concrete. This review synthesizes the durability and microstructural enhancements provided by bacterial concrete, with an emphasis on numerical improvements observed in various durability tests.

Bacterial self-healing in concrete predominantly utilizes ureolytic bacteria, including Bacillus subtilis and Bacillus pasteurii, to promote the precipitation of CaCO_3_ via MICP [1]. This biochemical mechanism transpires as the bacteria break down urea into ammonia and carbonate ions, which subsequently interact with calcium ions found in the concrete matrix, resulting in the formation of CaCO_3_ crystals that effectively seal microcracks and pores [2].

Studies have demonstrated that the incorporation of bacteria at optimal concentrations (10^6^ cells/ml) leads to a significant improvement in concrete properties. Specifically, bacterial concrete exhibits an increase in compressive strength by 15–25% compared to conventional concrete [3,4]. This enhancement is attributed to the densification of the microstructure due to CaCO_3_ precipitation, which enhances the bond between aggregate and cement paste. Furthermore, bacterial concrete significantly reduces water absorption by 30–45%, mitigating the ingress of harmful substances and improving durability. For instance, experimental results indicate that while conventional concrete recorded a water absorption rate of 5.53% at 28 days, bacterial concrete exhibited values as low as 3.50% [2,5] Beyond mechanical strength and reduced permeability, bacterial concrete offers potential advantages in sustainability and long-term performance. By enabling autonomous crack healing, it minimizes the need for maintenance and repair, reducing the consumption of raw materials and associated carbon emissions [6].

The incorporation of bacteria in concrete has been shown to significantly improve its durability by reducing permeability and increasing resistance to aggressive environmental conditions [7]. These enhancements stem from reductions in water absorption, gas permeability, and chloride ion diffusion, all of which contribute to the overall longevity of bacterial concrete [8]. For instance, RCPT results demonstrate that bacterial concrete containing Bacillus flexus at an optimal concentration of 10⁶ cells/ml reduces chloride ion penetration by approximately 42% [3,9]. Specifically, chloride ion diffusion values decreased from 2074 km/s in conventional concrete to 1202 km/s at 28 days, highlighting the ability of bacterial-induced calcite precipitation to create a denser microstructure and limit the ingress of harmful ions [10].

The sulphate resistance tests indicate that bacterial concrete exhibits significantly improved resistance to sulphate attack [11]. Weight loss measurements after sulphate exposure reveal that bacterial concrete reduces mass loss by up to 50%, with control concrete experiencing a mass loss of 5.54% at 28 days, while bacterial mixes recorded values as low as 2.73% [8,12]. This improvement is attributed to the formation of CaCO_3_, which fills microcracks and voids, thereby reducing pathways for sulphate ingress and subsequent deterioration. The results highlight the promise of bacterial self-healing concrete as a viable approach to improving the durability and longevity of concrete structures, especially in settings susceptible to chloride-induced corrosion and sulphate attack [13].

Bacterial activity in concrete leads to significant microstructural modifications, primarily through the precipitation of CaCO_3_, which forms calcite crystals that densify the concrete matrix [14]. These crystals, which appear in needle-like, bouquet-like, or rhombohedral morphologies, effectively fill microcracks and pores, enhancing the overall mechanical properties and durability of the concrete [9,15].

Advanced characterization techniques such as SEM and XRD have confirmed the formation of dense mineral deposits in bacterial concrete [2,6,16]. SEM analyses reveal a reduction in void ratio by up to 35% compared to conventional concrete, indicating a more compact microstructure with fewer pathways for water and harmful ions [12,17]. EDAX results have verified the presence of CaCO_3_, with calcium peaks in bacterial concrete being 1.5 times higher than those observed in conventional mixes [18]. These findings underscore the role of MICP in improving the microstructural integrity of concrete, leading to enhanced strength, reduced permeability, and increased resistance to environmental degradation [19].

The combined use of fly ash and silica fume in porous concrete can significantly enhance compressive strength and durability while maintaining an alkaline environment conducive to vegetation growth, supporting sustainable infrastructure applications [20].The effectiveness of bacterial concrete can be further enhanced by incorporating mineral additives such as natural zeolite, which interacts with bacterial precipitates to refine the microstructure and enhance durability [21]. These mineral additives contribute to additional pore refinement and improved binding of microbial-induced calcite deposits, leading to a denser and more resilient concrete matrix [22].

Utilizing rice husk ash as a bio-waste material in geopolymer composites with aluminum oxide can improve compressive strength and densify the matrix, promoting effective waste valorization in construction [23]. Moreover, the integration of pozzolanic materials such as fly ash and silica fume in combination with bacterial strains has been shown to yield an additional 10–15% enrich in axial strength. This enrichment is attributed to the synergetic effect of MICP and pozzolanic reactions, which together enhance the overall densification and hydration process [17,19,24]. These materials contribute secondary hydration products, filling voids and improving the interfacial transition zone, thereby increasing strength and durability [25]. Recent advances in genetic engineering have also been explored to optimize bacterial survival and self-healing efficiency within concrete. Genetically modified bacteria with bioremediase-like genes have demonstrated an extended lifespan within the concrete matrix and the ability to produce novel mineral phases, further improving structural integrity and longevity [19,26]. These engineered bacteria enhance CaCO_3_ precipitation rates and enable more effective self-healing, making bacterial concrete a promising solution for sustainable and long-lasting infrastructure [27]. Incorporating sisal fibers into geopolymer concrete increases tensile, compressive, and flexural strength, while also enhancing crack resistance and energy absorption, contributing to more ductile and durable cementitious materials [28].

Bacterial concrete, while offering numerous advantages, encounters several obstacles that need to be overcome for its broader implementation. A significant challenge is the elevated expense associated with bacterial cultures and nutrients, which can exceed that of traditional concrete additives by 20 to 30 percent [22,29]. This cost factor limits large-scale implementation and necessitates further research into cost-effective bacterial strains and nutrient alternatives. Another key challenge is ensuring bacterial viability under extreme environmental conditions, such as freeze-thaw cycles, carbonation exposure, and prolonged drying [18,30]. While laboratory studies have demonstrated the effectiveness of bacterial self-healing, real-world applications require further investigation to assess long-term performance in diverse climatic conditions. Strategies such as encapsulating bacteria in protective carriers, optimizing nutrient delivery systems, and selecting resilient bacterial strains are being explored to enhance durability and functionality [31].

Despite these challenges, bacterial concrete has shown superior strength and self-recuperating properties, particularly when using Bacillus subtilis JC3 microorganisms [32]. Adding polyvinyl alcohol and polypropylene fibers to cementitious composites reduces crack propagation and improves post-cracking behavior, leading to enhanced durability and resilience against aggressive environmental exposure [33]. Compared to traditional M25 concrete, bacterial concrete has been found to improve structural integrity and reduce crack width, thereby increasing service life and minimizing maintenance costs [34]. Continued advancements in microbial engineering, material optimization, and large-scale field studies will be critical in overcoming current limitations and paving the way for the broader adoption of this innovative and sustainable construction material [35]. The bacterial concrete's decreased porosity as a result of calcite precipitation was demonstrated by decreased water permeability (29–60%), chloride permeability (5–17%), and water absorption (3–32%) in comparison to the control specimen. It has been determined that Bacillus cereus GS-5 immobilized sintered fly ash lightweight aggregate would work well as an additive in concrete, offering benefits including improved mechanical performance, increased durability, and the capacity to self-heal fractures [36].

Limited Integration with Other Additives, while some studies mention pozzolans or zeolites, more comprehensive research is needed on the synergistic effects of bacterial action combined with various supplementary cementitious materials. Limited understanding exists on how bacteria survive over time within the harsh environment of concrete, especially under prolonged acidic conditions.

## Method details

The effectiveness of bacterial concrete is significantly influenced by the characteristics of the materials incorporated into its formulation, as each element is vital in establishing its mechanical strength, longevity, and ability to withstand environmental deterioration. Ordinary Portland Cement (OPC) of 33-grade, compliant with IS 12269:2013, was utilized as the main binding agent. The selection of OPC 33-grade is attributed to its superior early strength, which facilitates improved hydration and enhances the performance of bacterial concrete [37]. The chemical makeup of the cement, particularly the presence of tricalcium silicate (C_3_S) and dicalcium silicate (C_2_S), is vital for the hydration process, thereby affecting the overall strength development and durability of the concrete matrix.

Fine aggregate, sourced from locally available river sand (Zone II) in accordance with IS 383:2016, was utilized to achieve optimal gradation and workability. This fine aggregate exhibited a specific gravity of 2.65 and a fineness modulus of 2.8, demonstrating its appropriateness for the production of dense and workable concrete. The fine aggregate plays a crucial role by occupying the voids between cement particles, thereby minimizing porosity and enhancing the overall compactness of the concrete. Coarse aggregate, composed of crushed granite with a size of 20 mm, was employed to facilitate improved interlocking and load distribution [38]. The choice of crushed granite, recognized for its exceptional hardness and durability, contributes to the enhancement of both compressive and flexural strength of the concrete. The specific gravity of the coarse aggregate was recorded at 2.7, and it adhered to the standards set forth in IS 383:2016 to ensure uniformity in the mix.

Clean, potable water devoid of impurities was utilized for both mixing and curing, in accordance with the standards set forth in IS 456:2000. Water is essential in the hydration process, as it interacts with cement to produce calcium silicate hydrate (C-S-H) gel, which is crucial for the strength enhancement of concrete [39]. The water-to-cement ratio was carefully optimized to facilitate sufficient hydration while preventing excessive porosity, as increased porosity can undermine durability and elevate permeability [40]. EDAX involves directing an electron beam onto the sample surface to emit X-rays, identifying elemental composition through energy peaks corresponding to specific elements.

Bacillus subtilis is indeed one of the most extensively studied strains in MICP due to its urease activity and endospore formation, other strains were chosen to explore comparative performance and mechanisms under different metabolic conditions [41]. Pseudomonas stutzeri has been previously reported to facilitate calcite precipitation through denitrification pathways, offering a non-ureolytic route to MICP, which is advantageous in environments where ammonia accumulation is undesirable [42]. Escherichia coli, although not a natural MICP performer, is included due to its well-characterized genetics and potential as a chassis for synthetic biology applications aimed at enhancing MICP activity [43]. Other strains were selected based on prior studies or preliminary data suggesting urease production, biofilm formation, or environmental resilience relevant to biocementation processes [44].

The concrete was infused with various bacterial strains, namely Bacillus Licheniformis, Bacillus Flexus, Pseudomonas stutzeri, Escherichia coli, and Bacillus subtilis, chosen for their capacity to promote MICP. MICP is a natural biochemical mechanism whereby bacteria aid in the crystallization of CaCO_3_, effectively sealing cracks and voids within the concrete structure, thereby enhancing its impermeability and overall durability. The bacterial concentration varied from 10⁴ to 10⁶ cells/ml to evaluate its impact on mechanical and durability properties. Higher bacterial concentrations were found to enhance the self-healing capability and resistance to chloride penetration, sulphate attack, and acid degradation.

### Methodology

**Fig. 1:**
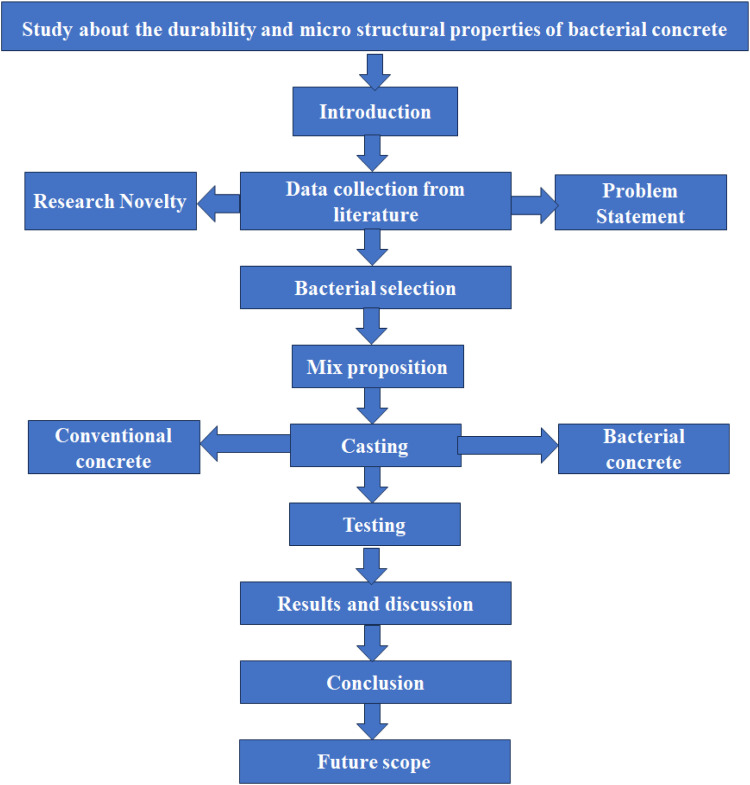
Methodology of the research work.

**Fig. 2:**
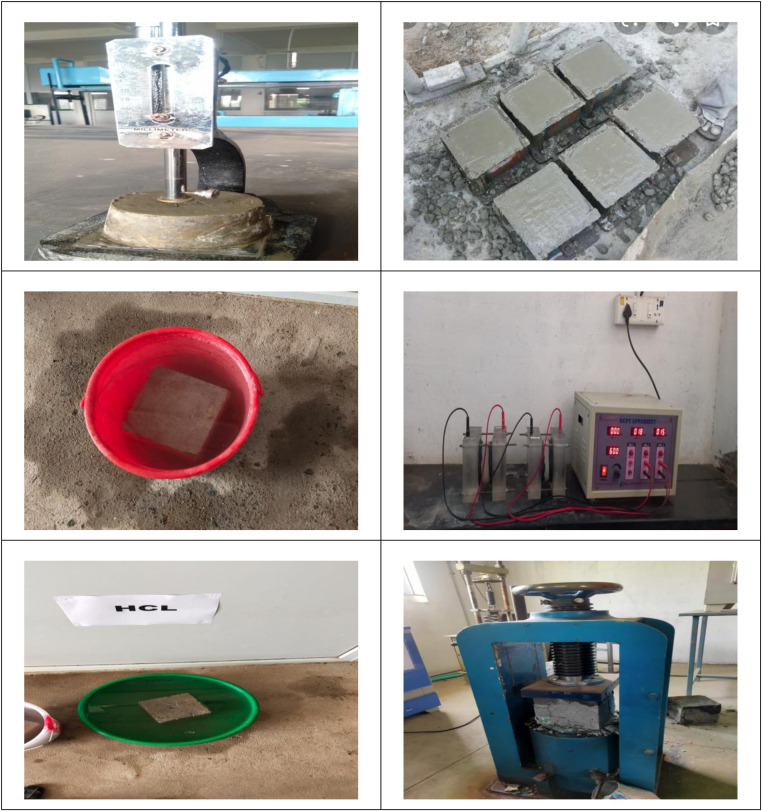
Experimental works.

Concrete casting involves preparing specimens for strength testing by pouring fresh concrete into molds of specified shapes and sizes. In this case, three types of specimens were cast: cubes, cylinders, and beams. The cube specimens, each measuring 15 cm × 15 cm × 15 cm, are primarily used for compressive strength tests. Cylindrical specimens with a diameter of 15 cm and a height of 30 cm are also used for splitting tensile strength. Beam specimens, with dimensions of 15 cm × 15 cm × 750 cm (subject to confirmation due to the unusually long length), are typically used for flexural strength testing. The concrete is poured into molds in layers and each layer is compacted using tamping rods or vibration to remove air voids. After casting, the top surface is levelled, and the specimens are kept covered for 24 hours to prevent moisture loss. They are then demolded and cured in water until the time of testing, typically at 7 or 28 days. Proper casting and curing are essential to ensure accurate and reliable test results. Fig. 1, Fig. 2 shows the methodology and experimental works of the research work. Table 1 show the mix designations of various mixes.

**Table 1:** Mix designations.

Mix No	Cement (kg/m^3^)	Flyash (kg/m^3^)	Fine aggregate (kg/m^3^)	Coarse aggregate (kg/m^3^)	Water (kg/m^3^)	Bacteria	Cell Concentration
M1	399.24	44.8	700	1040	201.62	-	-
M2	399.24	44.8	700	1040	201.62	Bacillus subtilis	10^4^
M3	399.24	44.8	700	1040	201.62		10^5^
M4	399.24	44.8	700	1040	201.62		10^6^
M5	399.24	44.8	700	1040	201.62	Bacillus Licheniformis	10^4^
M6	399.24	44.8	700	1040	201.62		10^5^
M7	399.24	44.8	700	1040	201.62		10^6^
M8	399.24	44.8	700	1040	201.62	Bacillus Flexus	10^4^
M9	399.24	44.8	700	1040	201.62		10^5^
M10	399.24	44.8	700	1040	201.62		10^6^
M11	399.24	44.8	700	1040	201.62	Pseudomonas stutzeri	10^4^
M12	399.24	44.8	700	1040	201.62		10^5^
M13	399.24	44.8	700	1040	201.62		10^6^
M14	399.24	44.8	700	1040	201.62	Escherichia Coli	10^4^
M15	399.24	44.8	700	1040	201.62		10^5^
M16	399.24	44.8	700	1040	201.62		10^6^

## Method validation

### Slump cone test

The slump test results demonstrate the impact of various bacterial strains on the workability of concrete. The control mix (M1) showed a slump of 110 mm. All bacterial concrete mixes exhibited increased slump values, indicating improved workability. In Bacillus subtilis mixes, slumps rose from 111 mm (M2, 10⁴ cells/ml) to 117 mm (M4, 10⁶ cells/ml). Bacillus licheniformis mixes showed a similar trend, with M5 to M7 ranging from 112 mm to 118 mm. Bacillus flexus-based mixes (M8–M10) ranged from 111 mm to 116 mm, confirming a consistent improvement. Pseudomonas stutzeri mixes (M11–M13) also followed the trend, increasing from 112 mm to 117 mm. Escherichia coli mixes (M14–M16) showed values from 112 mm to 117 mm, reinforcing bacterial influence on fresh concrete properties.

**Fig. 3:**
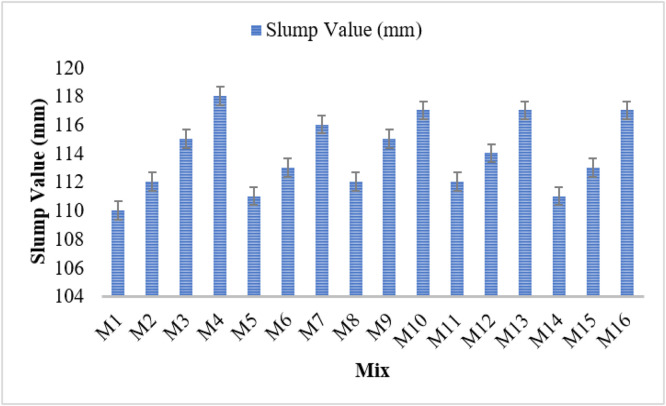
Slump cone results.

The observed increase in workability is attributed to microbial activity that enhances hydration and matrix structure. Bacteria promote nucleation sites and improve cement dispersion through extracellular polymeric substances (EPS) and bio-mineralization. Higher concentrations (≥10⁶ cells/ml) consistently improved workability. Among the strains, *Bacillus licheniformis* and *Pseudomonas stutzeri* had the most significant effect. These results suggest that bacterial concrete not only aids in self-healing but also improves fresh-state performance, making it a viable option for sustainable construction. Fig. 3 illustrates the slump cone results.

### Compressive strength test

The compressive strength results demonstrate the effectiveness of bacterial incorporation in enhancing both early-age strength and self-healing capacity of concrete. The control mix (M1) exhibited a 28-day strength of 24.03 MPa, with moderate strength recovery after cracking. Bacterial concrete mixes showed improved performance, with strength increasing proportionally with bacterial concentration.

Among all strains, *Bacillus subtilis* (M14–M16) achieved the highest strength, reaching 28.85 MPa at 28 days for M16 (10⁶ cells/ml), and a healed strength of 31.45 MPa at 40 days. Other strains, including *Bacillus licheniformis, Bacillus flexus, Pseudomonas stutzeri*, and *Escherichia coli*, followed a similar trend, with increased strength and self-healing as cell concentration increased. Notably, mixes with 10⁶ cells/ml consistently outperformed lower concentrations and the control.

**Fig. 4:**
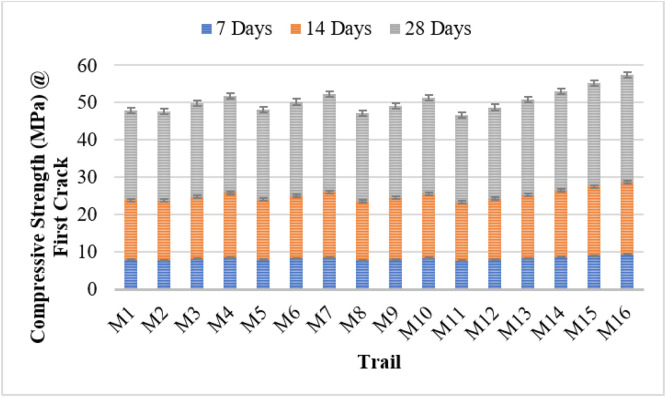
Compressive strength of first crack.

**Fig. 5:**
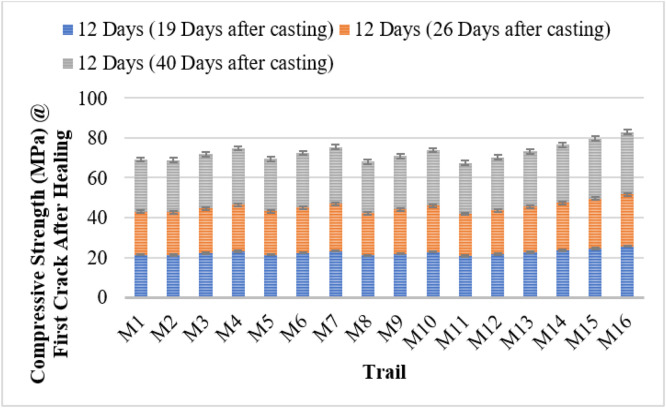
Compressive strength of first crack after healing.

Self-healing behavior was evident through the progressive recovery in strength post-cracking, particularly between 19 and 40 days. The bacterial activity promoted calcium carbonate precipitation, aiding crack sealing and strength restoration. These findings confirm that bacterial concrete not only improves early mechanical properties but also extends the service life of concrete through autonomous healing. The use of *Bacillus subtilis* is especially promising for sustainable and durable construction. Fig. 4, Fig. 5 shows the compressive strength of first crack and first crack after healing.

### Saturated water absorption test

The findings reveal that the integration of bacterial strains into concrete markedly decreases its saturated water absorption over time when compared to traditional concrete (M1). The control mix (M1) showed the greatest absorption rates, recording values of 2.96%, 2.69%, and 2.42% at 28, 56, and 90 days, respectively. In contrast, the bacterial concrete mixtures exhibited reduced water absorption, underscoring the efficacy of microbial-induced calcium carbonate precipitation (MICP) in enhancing the durability of concrete.

Among the different bacterial strains used, Bacillus Licheniformis (M2–M4) exhibited a decreasing trend in water absorption with increasing cell concentrations. M4 (10^6^ cells/ml) showed the lowest absorption values of 1.72%, 1.57%, and 1.41% at 28, 56, and 90 days, respectively. A similar trend was observed for Bacillus Flexus (M5–M7), where M7 recorded the lowest absorption of 1.62%, 1.47%, and 1.32%, indicating enhanced densification of the concrete matrix.

Pseudomonas stutzeri (M8–M10) and Escherichia coli (M11–M13) also exhibited reduced absorption values with increasing bacterial concentration. The lowest values recorded were 1.73%, 1.58%, and 1.41% for both bacterial strains at 106 cells/ml (M10, M13). Similarly, Bacillus subtilis (M14–M16) showed a significant reduction in absorption, with M16 demonstrating the lowest values of 1.66%, 1.51%, and 1.35%.

**Fig. 6:**
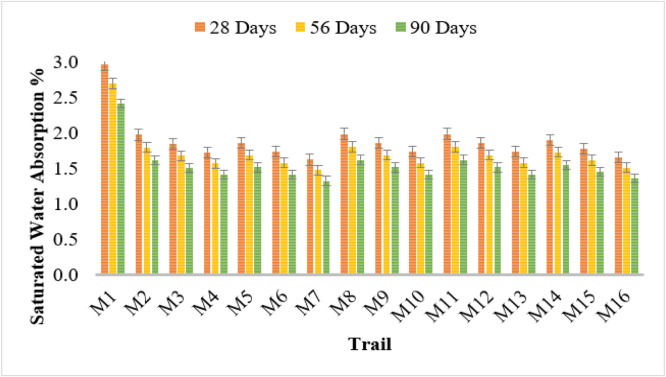
Saturated water absorption test results.

The reduction in water absorption can be attributed to the microbial-induced precipitation of calcium carbonate, which enhances the compactness and impermeability of concrete [24] The effectiveness of bacterial strains increases with higher concentrations, and Bacillus Flexus and Bacillus Licheniformis at 10^6^ cells/ml exhibited the most significant improvements. These findings suggest that microbial concrete can enhance durability and reduce permeability, making it a viable alternative to conventional concrete. Fig. 6 shows the saturated water absorption test results.

### Rapid chloride permeability test (RCPT)

The RCPT results indicate that bacterial concrete exhibits a significant reduction in chloride ion penetration compared to conventional concrete (M1). The control mix (M1) displayed the highest RCPT values, with readings of 2074, 1855, and 1727 km/s at 28, 56, and 90 days, respectively, indicating higher permeability.

Among bacterial strains, Bacillus Licheniformis (M2–M4) showed a decline in RCPT values with increasing bacterial concentration. M4 (10^6^ cells/ml) demonstrated the lowest chloride penetration at 1322, 1183, and 1101 km/s over the respective curing periods. Similarly, Bacillus Flexus (M5–M7) exhibited a similar trend, with M7 recording the lowest values of 1202, 1075, and 1001 km/s, confirming its effectiveness in enhancing concrete impermeability.

Pseudomonas stutzeri (M8–M10) and Escherichia coli (M11–M13) also reduced chloride penetration significantly. The lowest values for both strains at 10^6^ cells/ml (M10, M13) were 1352, 1209, and 1126 km/s, and 1460, 1306, and 1216 km/s, respectively. Bacillus subtilis (M14–M16) demonstrated the most substantial improvement in reducing chloride permeability, with M16 showing the lowest values of 1041, 931, and 867 km/s.

**Fig. 7:**
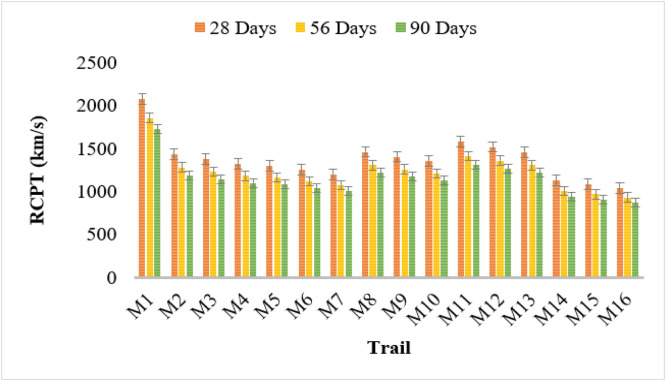
RCPT test results.

These findings suggest that microbial concrete can effectively enhance resistance to chloride penetration, reducing the risk of reinforcement corrosion. The use of Bacillus Flexus, Bacillus Licheniformis, and Bacillus subtilis at higher concentrations provides the most substantial improvement, making bacterial concrete a promising solution for durable and long-lasting. Fig. 7 shows the RCPT test results.

### Sulphate attack test

The sulphate resistance analysis reveals that bacterial concrete significantly reduces the percentage loss of weight due to sulphate attack, both before and after healing. Conventional concrete (M1) displayed the highest weight loss values at 5.54%, 5.01%, and 4.85% at 28, 56, and 90 days, respectively, indicating lower resistance to sulphate attack.

Among bacterial strains, Bacillus Licheniformis (M2–M4) showed a reduction in weight loss with increasing bacterial concentration. M4 (106 cells/ml) exhibited the lowest weight loss of 3.43%, 3.10%, and 3.00% over the curing periods. Furthermore, after healing, the weight loss further reduced to 2.41%, 2.18%, and 2.11%, demonstrating improved self-healing capabilities.

Similarly, Bacillus Flexus (M5–M7) demonstrated a notable decrease in weight loss due to sulphate attack. M7 (10^6^ cells/ml) recorded the lowest values of 2.84%, 2.56%, and 2.48% before healing, which further decreased to 1.99%, 1.80%, and 1.74% after healing. Pseudomonas stutzeri (M8–M10) and Escherichia coli (M11–M13) also contributed to enhanced sulphate resistance. The lowest recorded values for Escherichia coli at 10^6^ cells/ml (M13) were 3.65%, 3.30%, and 3.19% before healing, which further improved to 2.56%, 2.32%, and 2.24% after healing.

Among all bacterial strains, Bacillus subtilis (M14–M16) showed the highest sulphate resistance, with M16 recording the lowest weight loss values of 2.73%, 2.47%, and 2.39% before healing, further reducing to 1.91%, 1.73%, and 1.68% after healing.

**Fig. 8:**
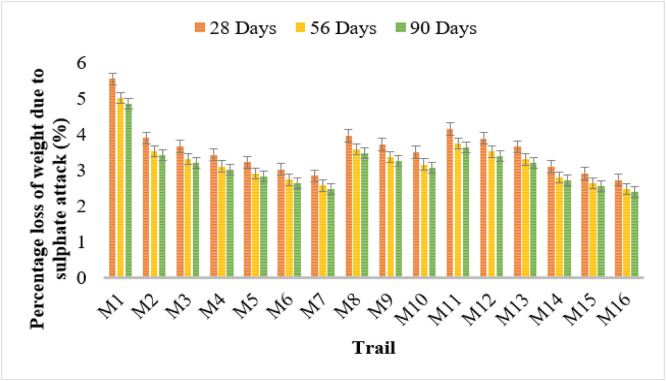
Loss of weight in sulphate attack.

**Fig. 9:**
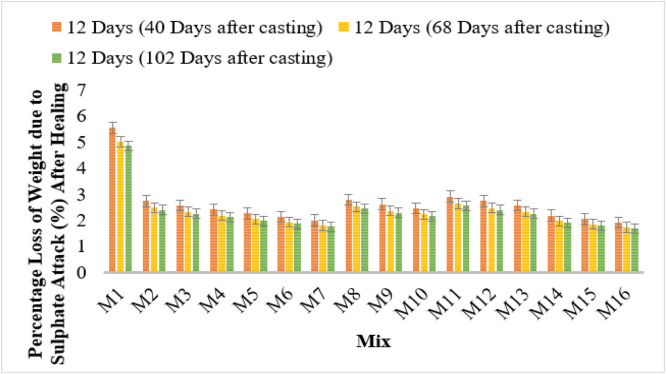
loss of weight in sulphate attack after healing test results.

These results confirm that microbial concrete, particularly with higher bacterial concentrations, significantly enhances sulphate resistance. The reduction in weight loss after healing demonstrates the effectiveness of microbial self-healing, making bacterial concrete an optimal choice for sulphate-exposed environments. Fig. 8, Fig. 9 shows the sulphate attack test results.

### Acid resistance test

The hydrochloric acid resistance test results demonstrate that bacterial concrete enhances acid resistance by reducing weight loss over time. Conventional concrete (M1) exhibited the highest weight loss, with values of 6.23%, 5.64%, and 5.46% at 28, 56, and 90 days, respectively.

Among bacterial strains, Bacillus Licheniformis (M2–M4) showed a decline in weight loss with increasing bacterial concentration. M4 (10^6^ cells/ml) displayed the lowest values of 4.32%, 3.91%, and 3.78%. Similarly, Bacillus Flexus (M5–M7) exhibited improved resistance, with M7 showing the lowest weight loss of 3.57%, 3.23%, and 3.13%.

Pseudomonas stutzeri (M8–M10) and Escherichia coli (M11–M13) demonstrated similar improvements. The lowest recorded values for Escherichia coli at 10^6^ cells/ml (M13) were 4.60%, 4.16%, and 4.02%. Among all bacterial strains, Bacillus subtilis (M14–M16) showed the highest acid resistance, with M16 recording the lowest weight loss values of 3.43%, 3.11%, and 3.01%. These findings indicate that microbial concrete enhances acid resistance, making it suitable for environments exposed to acidic conditions.

The results indicate that bacterial concrete significantly reduces the percentage loss of strength due to hydrochloric acid exposure. Conventional concrete (M1) displayed the highest strength loss values at 7.02%, 6.35%, and 6.15% at 28, 56, and 90 days, respectively, indicating lower resistance to acid attack.

**Fig. 10:**
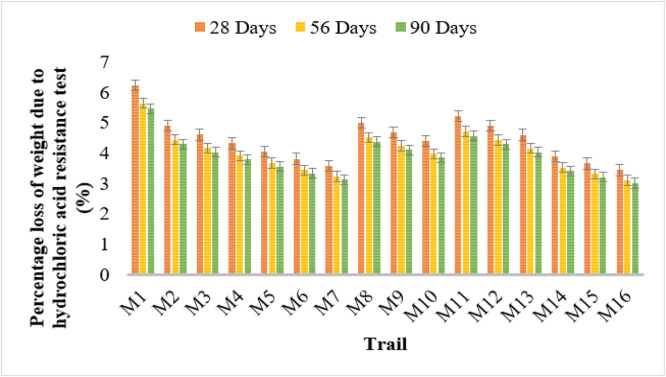
Weight loss in acid resistance test results.

**Fig. 11:**
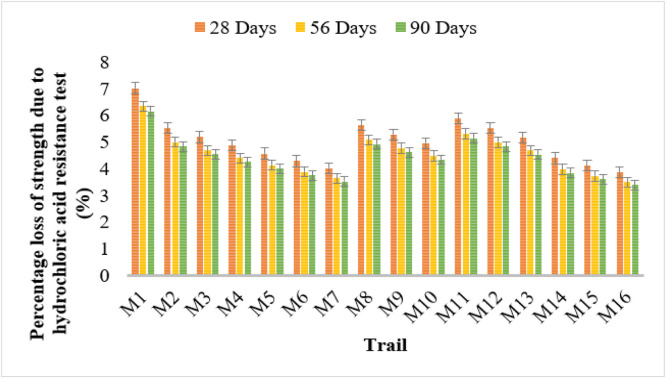
Strength loss in acid resistance test results.

Among bacterial strains, Bacillus Licheniformis (M2–M4) exhibited a decreasing trend in strength loss with increasing bacterial concentration. M4 (10^6^ cells/ml) showed the lowest strength loss values of 4.87%, 4.40%, and 4.26% over the curing periods. Similarly, Bacillus Flexus (M5–M7) demonstrated notable improvements, with M7 recording the lowest values of 4.03%, 3.64%, and 3.52%. Bacillus subtilis (M14–M16) showed the most substantial resistance, with M16 recording the lowest strength loss values of 3.87%, 3.50%, and 3.39%, demonstrating its superior acid resistance. Fig. 10, Fig. 11 shows the weight and strength loss in acid resistance test results.

### Sorptivity test

Sorptivity serves as an essential metric that reflects the speed at which capillary water is absorbed by concrete. The findings reveal that bacterial concrete shows markedly lower sorptivity values in comparison to traditional concrete (M1), highlighting the efficacy of microbial-induced calcium carbonate precipitation (MICP) in diminishing permeability.

The control mix (M1) exhibited the highest sorptivity values at 5.53 × 10^-5^ m/s¹/², 5.00 × 10^-5^ m/s¹/², and 4.84 × 10^-5^ m/s¹/² at 28, 56, and 90 days respectively. The addition of bacterial strains led to a notable reduction in sorptivity, with the effect being more pronounced at higher bacterial concentrations.

Among the different bacterial strains, Bacillus Licheniformis (M2–M4) showed a decreasing trend in sorptivity with increasing bacterial concentration. M4 (10⁶ cells/ml) recorded the lowest values within this strain group at 3.67 × 10^-5^ m/s¹/², 3.32 × 10^-5^ m/s¹/², and 3.22 × 10^-5^ m/s¹/² at 28, 56, and 90 days respectively. A similar trend was observed for Bacillus Flexus (M5–M7), where M7 exhibited the lowest sorptivity values of 3.50 × 10^-5^ m/s¹/², 3.16 × 10^-5^ m/s¹/², and 3.06 × 10^-5^ m/s¹/² at the same time intervals.

The effect of Pseudomonas stutzeri (M8–M10) was also significant, with M10 (10⁶ cells/ml) showing lower sorptivity values of 3.85 × 10^-5^ m/s¹/², 3.48 × 10^-5^ m/s¹/², and 3.37 × 10^-5^ m/s¹/² at 28, 56, and 90 days. Escherichia coli (M11–M13) followed a similar trend, with the lowest sorptivity observed in M13 at 3.87 × 10^-5^ m/s¹/², 3.50 × 10^-5^ m/s¹/², and 3.39 × 10^-5^ m/s¹/².

Among all bacterial strains, Bacillus subtilis (M14–M16) demonstrated the most significant reduction in sorptivity. M16 (10^6^ cells/ml) achieved the lowest values recorded in the study: 3.62 × 10^-5^ m/s¹/², 3.27 × 10^-5^m/s¹/², and 3.17 × 10^-5^m/s¹/² at 28, 56, and 90 days respectively.

**Fig. 12:**
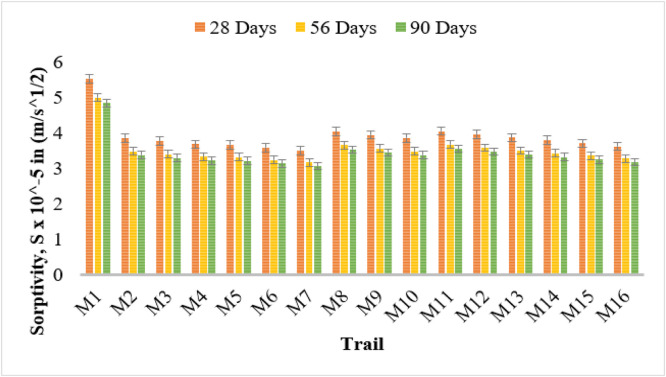
Sorptivity results showing reduced water absorption in bacterial concrete mixes compared to conventional concrete.

The overall reduction in sorptivity with bacterial addition can be attributed to the precipitation of calcium carbonate within the pores, leading to increased densification of the concrete matrix. The decreasing trend over time indicates the continued effectiveness of bacterial healing and densification. These findings confirm that microbial concrete enhances water resistance and durability, making it a viable alternative to conventional concrete in moisture-prone environments. Fig. 12 shows the sorptivity test results.

### EDAX

Energy Dispersive X-ray Analysis (EDAX) was conducted on specific concrete mixtures (M1 and M16) to investigate their elemental composition and confirm the occurrence of microbial-induced calcium carbonate precipitation (MICP). The analysis primarily aimed to identify calcium (Ca), oxygen (O), carbon (C), and other critical elements that play a role in enhancing the densification and durability of microbial concrete.

The EDAX spectrum of M1 (control mix) indicated a relatively lower calcium (Ca) content and a higher percentage of silicon (Si) and oxygen (O), signifying a porous and less dense structure. The limited presence of carbon (C) suggests minimal calcium carbonate (CaCO_3_) deposition, which correlates with the higher water absorption, chloride penetration, and sorptivity values observed in the durability tests.

M16 (10^6^ cells/ml of Bacillus subtilis) exhibited the highest calcium (Ca) and carbon (C) concentrations among all bacterial concrete mixes, indicating the most substantial calcium carbonate precipitation. The oxygen (O) content was significantly lower, confirming the most compact and impermeable microstructure. This aligns with M16′s lowest recorded values for sorptivity, chloride penetration, and sulphate resistance, making it the most durable mix in this study.

The EDAX analysis confirms that bacterial concrete enhances durability by promoting calcium carbonate precipitation, which refines the pore structure and reduces permeability. The microbial activity leads to increased calcium (Ca) and carbon (C) levels, while reducing oxygen (O) and silicon (Si) content, indicating a denser and more impermeable matrix. Among the studied Bacillus subtilis (M16) exhibited the most significant improvements, reinforcing their effectiveness in enhancing concrete durability. These findings validate the role of microbial-induced self-healing in prolonging the lifespan of concrete structures exposed to aggressive environmental conditions. EDAX data confirms the formation of CaCO₃; however, it cannot differentiate between polymorphs of calcium carbonate or verify crystallinity. Fig. 13 shows the EDAX test results (Fig. 13b).

**Fig. 13a:**
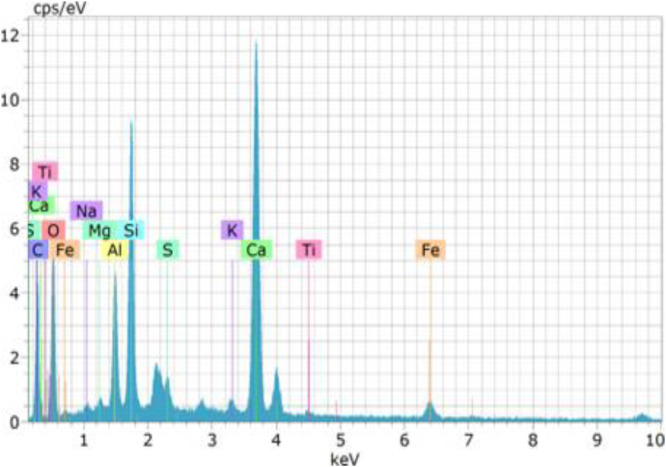
EDAX of M1 mix.

**Fig. 13b:**
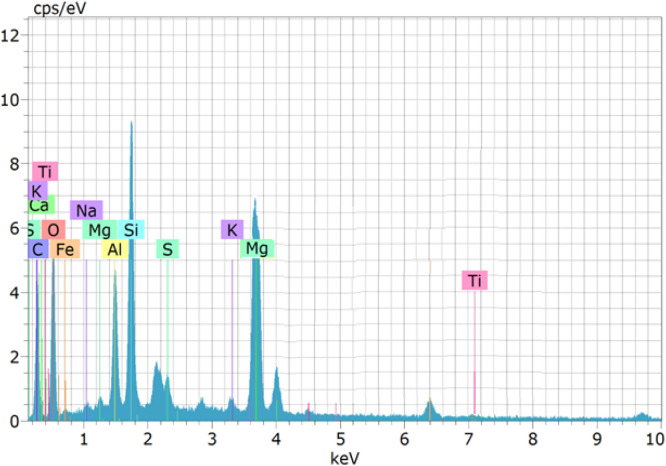
EDAX of M16 mix. EDAX test results.

## Conclusion

The experimental investigation on bacterial concrete incorporating different microbial strains (Bacillus Licheniformis, Bacillus Flexus, Pseudomonas stutzeri, Escherichia coli, and Bacillus subtilis) has demonstrated significant improvements in durability properties compared to conventional concrete. The results from water absorption, chloride penetration (RCPT), sulphate resistance, hydrochloric acid strength loss, sorptivity, and EDAX analysis confirm the effectiveness of MICP in enhancing concrete performance.

The water absorption test indicated that bacterial concrete exhibited significantly lower absorption values than conventional concrete. Among the bacterial strains, Bacillus Flexus (M7) and Bacillus Licheniformis (M4) at 10^6^ cells/ml showed the lowest water absorption values, highlighting their ability to refine the pore structure and improve the matrix density. This directly correlates with the observed reductions in permeability and improved resistance to external aggressive agents.

RCPT results further validated the enhancement in impermeability, with bacterial concrete mixes showing a substantial decline in chloride penetration. Bacillus subtilis (M16), followed by Bacillus Flexus (M7), exhibited the lowest chloride ion penetration values, confirming their superior resistance to corrosion-inducing agents. This suggests that microbial concrete can significantly extend the lifespan of reinforced concrete structures, especially in chloride-laden environments.

The sulphate resistance test results indicated that bacterial concrete showed reduced weight loss due to sulphate attack, both before and after self-healing. The lowest weight losses were recorded for Bacillus subtilis (M16) and Bacillus Flexus (M7), demonstrating their superior sulphate resistance and self-healing capabilities. The reduction in weight loss after the healing process suggests that bacterial concrete can autonomously repair microcracks, further enhancing its durability in aggressive environments.

Hydrochloric acid strength loss and sorptivity tests also confirmed that bacterial concrete offers improved resistance to acid attack and reduced capillary water absorption. Sorptivity results showed that M16, M7, and M4 exhibited the lowest absorption rates, indicating a highly compact and dense matrix, thereby reducing the ingress of harmful substances.

Finally, the EDAX analysis confirmed the presence of calcium carbonate (CaCO3) precipitation in bacterial concrete, which contributes to pore refinement and improved durability. The highest calcium (Ca) and carbon (C) contents were observed in M16 verifying their superior MICP activity and enhanced matrix densification.

Microbial concrete significantly enhances durability by reducing permeability, improving resistance to chloride penetration and sulphate attack, and promoting self-healing. The findings suggest that Bacillus subtilis, Bacillus Flexus, and Bacillus Licheniformis at higher concentrations (106 cells/ml) are the most effective in improving concrete performance, making bacterial concrete a promising alternative.

## Future scope

Future work focusses on optimizing multi-nutrient systems that sustain bacterial activity for extended periods, reducing overall costs, and enhancing the eco-friendliness of bacterial concrete. Investigating alternative bacterial strains with higher calcite production efficiency and developing bio-based carrier materials for improved bacterial survival could further enrich the effectiveness of self-healing concrete. By integrating microbial concrete into self-healing structural systems, such as encapsulation of bacteria in lightweight aggregates or microcapsules, to automate crack repair over the service life of structures. Comprehensive life-cycle assessments and cost–benefit analyses are necessary to establish the economic and environmental viability of microbial concrete as a sustainable construction material.

## Limitations

Not applicable

## Ethics statements

The paper reflects the authors' own research and analysis in a truthful and complete manner.

## CRediT authorship contribution statement

**Anumol Sukumaran:** Conceptualization, Methodology, Writing – original draft. **V Johnpaul:** Conceptualization, Investigation, Data curation, Writing – review & editing. **N Balasundaram:** Resources, Supervision, Project administration, Writing – review & editing. **S Senthil Kumar:** Visualization, Data curation, Writing – review & editing.

## Declaration of competing interest

The authors declare that they have no known competing financial interests or personal relationships that could have appeared to influence the work reported in this paper.

## Data Availability

Data will be made available on request.
